# Durian Fruits Discovered as Superior Folate Sources

**DOI:** 10.3389/fnut.2018.00114

**Published:** 2018-11-28

**Authors:** Lisa Striegel, Soraya Chebib, Caroline Dumler, Yuyun Lu, Dejian Huang, Michael Rychlik

**Affiliations:** ^1^Chair of Analytical Food Chemistry, Technical University of Munich, Freising, Germany; ^2^Department of Chemistry, Food Science and Technology Programme, National University of Singapore, Singapore, Singapore; ^3^Centre for Nutrition and Food Sciences, Queensland Alliance for Agriculture and Food Innovation, The University of Queensland, Brisbane, QLD, Australia

**Keywords:** stable isotope dilution assay, LC-MS/MS, folate, durian, tropical fruit

## Abstract

Durian (*Durio zibethinus*) is a tropical fruit grown in Southeast Asia and highly appreciated by consumers throughout Asia. Folates are a group of vitamins and are essential nutrients for humans. Here we present the folate analysis of different durian cultivars as well as diverse durian products. An LC-MS/MS method and the application of a stable isotope dilution assay (SIDA) was used for quantitation of the folate vitamers 5-methyltetrahydrofolate, 5-formyltetrahydrofolate, 10-formylfolate, tetrahydrofolate, and pteroylmonoglutamic acid by using [^13^C_5_]-labeled internal standards. Total folates varied from 175 to 440 μg/100 g for durian arils and from 15.0 to 417 μg/100 g for durian products. These contents are extraordinarily high compared to other fruits and may be correlated to the upregulated methionine biosynthesis pathway reported recently. In summary, the tropical fruit durian can be considered as a very rich dietary source of natural folates.

## Introduction

The Durian (*Durio zibethinus*) is a tropical fruit and has been cultivated for centuries in Southeast Asia. Durian is so special that the naturalist Alfred Russel Wallace once wrote about durian: “to eat durian is a new sensation worth a voyage to the East to experience.” Due to Durian's strong sulfury odor, almost everyone from Western countries first experiencing this smell is disgusted, but in Southeast Asia and major parts of China, durian is considered “the king of fruits”. Durian's high appreciation is mainly attributable to the creamy sweet taste of durian pulp, which is multiply used in beverages and desserts. Durian's aril is usually eaten fresh or freeze dried, but can also be processed and used for jam, candies or durian cake.

Therefore, “the king of fruits“ is one of the most prized Southeast Asian crops and gained substantial economic value particularly due to durian exports to China exceeding 600 million US$ in 2016; even Singapore imported durians at a trade value of over 8 million US$ in the same year (1). In Southeast Asia an estimation of the durian contribution to the dietary supply of folates can be performed as follows. Current estimation for the consumption in the countries with highest durian intake per capita and year are 14, 13, and 10.25 kg in Thailand, Singapore and Malaysia, respectively (2). When assuming the edible aril constituting about 25% of the whole fruit (2), the mean consumption of Durian arils in Malaysia would be ~7 g per day.

Whereas, the sulfur containing impact odorants already have been elucidated as ethanethiol, 1-(ethylsulfanyl)ethane-1-thiol, methanethiol and ethane-1,1-dithiol (3) and recent whole genome sequencing (4) revealed sulfur and particular γ-methionine lyase related genes to be responsible for its odor, the nutritive value of durian is largely unknown or less acknowledged. There is essentially one review that compiled the nutrient contents in durian and revealed no particular abundance of essential nutrients or micronutrients (5). However, the vitamin analysis reviewed in the latter were restricted to vitamin C, thiamin, riboflavin, niacin, pantothenic acid, vitamin A, and beta carotene. In particular, the content of one of the most critical vitamin group, the folates, needs further examination.

The group of folates plays an essential role in metabolic pathways involving one-carbon transfers essential in nucleotide biosynthesis and remethylation of homocysteine to methionine (6, 7). As humans are not able to synthesize these compounds, a sufficient supply has to be warranted with the diet or supplements. However, in countries without mandatory folate fortification, dietary folate intake has been reported to cover the recommended requirements only partly. In this regard, a lack of folate is mainly correlated with neural tube defects in newborns (8) but can also result in increased levels of homocysteine being associated with cardiovascular diseases (9), and Alzheimer‘s disease (10). In contrast to the US and almost the whole Americas with mandatory folate fortification being in place, major regions in Europe are supposed to be in folate deficiency (11). Also in China, in particular the population of northern regions is known to be marginally supplied with dietary folate (12). These indispensable functions and frequent shortcomings in folate supply underline the importance of reliable analysis of folates in foods important for diets in specific regions.

Some particular challenges of folate analysis arise from their high number of often interconverting vitamers, their susceptibility to oxygen, elevated temperature and light and their occurrence only in traces. As compared to other methodologies such as microbiological assays, the application of stable isotope dilution assay (SIDA) has major advantages including the complete compensation for losses of analytes during extraction and for ion suppression during LC-MS/MS and providing information on the folate vitamer pattern (13).

With durian being an important fruit in Southeast Asia and China, we report herein the quantitation of the main folate vitamers pteroylmonoglutamic acid (PteGlu), tetrahydrofolate (H_4_folate), 5-methyltetrahydrofolate (5-CH_3_-H_4_folate), 10-formylfolate (10-CHO- PteGlu), and 5-formyltetrahydrofolate (5-CHO-H_4_folate) in different durian cultivars from Malaysia, Thailand and Indonesia. Moreover, as durian is processed to several other products on the market, we analyzed also regionally popular durian products.

## Materials and methods

### Samples and study design

A diverse set of popular and indigenous durian varieties was investigated in the present study. One single fruit of different durian types belong them King of the king, Black Thorn (Ochee), Musang King, D24 (Sultan), Black pearl (Hei Zhen Zhu), Red prawn (Hong Xia, Ang Hay), Golden phoenix (Jin Feng), D101 as well as one unknown type were bought from a fruit market in Singapore and prepared for transport and analysis from the Department of Chemistry, National University of Singapore, Singapore. The fruits were harvested at the eating ripe stage. The edible part (durian flesh) was cut into longer stripes for the determination of the moisture content. The moisture content was determined in triplicates using a UniBloc Moisture Analyzer (Shimadzu, Kyoto, Japan). After drying the durian samples were vacuumized and sent to the Institute of Analytical Food Chemistry, Technical University of Munich, Freising, Germany.

A further durian cultivar Mon Thong (Golden pillow), originally from Vietnam, was purchased on a local market (Viktualienmarkt, München). The edible durian flesh was cut into pieces and freeze dried using a freeze dryer Alpha 1-2 LDplus (Christ, Osterode am Harz, Germany). The moisture content was determined in triplicate.

Furthermore, commercially manufactured durian products, among them durian chips “Durian Monthong, Fruit King” (ingredients: 100% durian without additives) (Sunshine International CO., LTD., Chanthaburi, Thailand), “Durian Chunk” (ingredients: 100% durian without additives) (Fancyworld CO., LTD., Suphanburi, Thailand), “Kitshio Durian Chips”(ingredients: 100% durian without additives) (Onec Enterprise CO., LTD., Chanthaburi, Thailand), and durian paste “Monthong durian paste” (ingredients: 90% durian, 10% sugar) (sealed product from Bangkok, Thailand) were purchased in a commercial supermarket in Singapore and also sent to the Institute.

### Chemicals

Water, methanol and acetonitrile (LC-MS/MS grade and HPLC grade) were obtained from VWR (Ismaning, Germany); ascorbic acid, formic acid (>95%) and 2-(N-morpholino)-ethanesulfonic acid (MES) were purchased from Sigma-Aldrich (Steinheim, Germany); potassium dihydrogen phosphate, sodium acetate trihydrate and sodium hydroxide from Merck (Darmstadt, Germany); disodium hydrogen phosphate (anhydrous) and sodium chloride from Alfa Aesar and Baker J.T. (Thermo Fisher, Karlsruhe, Germany); rat serum and chicken pancreas containing γ-glutamyl hydrolase (EC 3.4.19.9) from Biozol (Eching, Germany) and Difco (Sparks, MD, United States), and DL-dithiothreitol (DTT) was aquired from Applichem (Darmstadt, Germany).

The unlabeled reference compounds (H_4_folate, 5-CH_3_-H_4_folate, 5-CHO-H_4_folate and 10-CHO-PteGlu) as well as the isotopological internal standards ([^13^C_5_]-PteGlu, [^13^C_5_]-H_4_folate, [^13^C_5_]-5-CH_3_-H_4_folate, and [^13^C_5_]-5-CHO-H_4_folate) were purchased from Schircks Laboratories (Jona, Switzerland), whereas PteGlu was obtained from Fluka (Sigma-Aldrich, Steinheim, Germany). Strata SAX cartridges (quaternary amine, 500 mg, 3 mL) were bought from Phenomenex (Aschaffenburg, Germany).

### Solutions

Buffer used for extraction consisting of 200 nmol/L 2-(N-morpholino)ethanlsulfonic acid hydrate (MES), 114 nmol/L ascorbic acid and 0.7 nmol/L DTT with a pH adjusted to 5.0. Phosphate buffer used for dissolving the unlabeled reference compounds and used for preparing the solution for elution, was prepared by dissolving 100 mmol/L sodium hydrogen phosphate and adjusting the solution with 100 mmol/L dipotassium hydrogen phosphate to pH 7.0. For equilibrating the SPE cartridges, 10 mmol/L phosphate buffer was mixed with 1.3 mmol/L DTT. For eluting the analytes during SPE extraction, a buffer consisting of 5% sodium chloride, 1% ascorbic acid, 100 mmol/L sodium acetetate, and 0.7 mmol/L DTT. For deconjugation, chicken pancreas consisting of 1 g/L chicken pancreas dissolved in 100 mmol/L phosphate buffer and 1% ascorbic acid was adjusted to pH7. Rat serum was used without further dilution.

### Stock solutions of analytes and internal standards

For each extraction, stock solutions of the reference material were prepared by first dissolving 10 mg of PteGlu in 100 mL of MES, pre-dissolved in 10 mL of phosphate buffer and 2 mg of H_4_folate, 5-CH_3_-H_4_folate, 5-CHO-H_4_folate, and 10-CHO-PteGlu in 10 mL MES, presolved in 3 mL of phosphate buffer. The purity and concentration of the unlabeled analytes was determined by HPLC/DAD using PteGlu as internal standard. The stock solutions were diluted 1:20 for the LC-MS/MS measurements. The isotopological internal standards [^13^C_5_]-PteGlu, [^13^C_5_]-H_4_folate, [^13^C_5_]-5-CH_3_-H_4_folate and [^13^C_5_]-5-CHO-H_4_folate were once dissolved in concentrations of 60–70 μg/mL in extraction buffer. For the sample extraction the internal standards were further diluted to final concentrations of 8–13 μg/mL suitable for addition during extraction. All reference solutions were stored at −20°C in the dark. The concentration of the labeled reference material was determined during each extraction preparing a response mix of labeled and unlabeled reference material.

### Sample preparation

The sample extraction was performed as published recently (14). Briefly, initially homogenized food samples (50 mg of durian on dried weight basis, 50 mg of durian products on dried weight basis) were weighed into Pyrex bottles and 10 mL of buffer was added. The mixtures were equilibrated for 15 min. The samples were spiked with internal standards in amounts adjusted to the expected contents of the respective analytes to fall in the given calibration range. Then, the samples were equilibrated again for 15 min and boiled for further 10 min. Once the samples were cooled on ice, 2 mL chicken pancreas suspension and 0.8 mL rat serum (amount of enzymes adjusted to receive complete deconjugation) were added and the mixture was incubated overnight (with a minimum of 12 h). Further, the samples were boiled for 10 min, cooled on ice, transferred into centrifuge tubes with 10 mL acetonitrile and centrifuged for 20 min (4,000 rpm, 4°C). After centrifugation, the supernatant with the aqueous and organic phase (~20 mL) was purified by strong anion-exchange (SAX) solid-phase extraction (SPE). The SAX-cartridges (quaternary amine, 500 mg, 3 mL) were first conditioned with two volumes of methanol and equilibrated with two volumes of buffer. After applying the extracts completely to the cartridges, the cartridges were washed with 3 volumes of equilibration buffer and run dry. The folates were eluted into test tubes using 2 mL elution buffer and the cartridges were run dry again. The eluate was membrane filtered (PVDF, 0.22 μm) and measured by LC-MS/MS.

### Instrumental conditions

The analysis was performed using the previously published instrumental conditions (14). The concentration of the solutions of unlabeled analytes, which were prepared new before each extraction, was performed on a Shimadzu HPLC/DAD system (Shimadzu, Kyoto, Japan) equipped with a reversed phase column (C18 EC, 250 × 3 mm, 5 μm, 100 Å, precolumn: C18, 8 × 3 mm, Machery-Nagel, Düren, Germany). The mobile phase for gradient elution consisted of a mixture of (A) 0.1% acetic acid and (B) methanol with a flow rate of 0.4 mL/min. Gradient elution started at 10% B for 7 min, followed by raising the concentration of B linearly to 50% within 14 min. Then, the gradient linearly went up to 100% B within 2 min, and then held at 100% B for 1 min. Finally, the mobile phase returned to the initial mixture of 10% B within 2 min and was equilibrated for 9 min before the next run. The injection volume was 10 μL and the analysis was done at room temperature. Data acquisition was performed with LabSolutions software 5.71 (Shimadzu, Kyoto, Japan).

LC-MS/MS was carried out on a Shimadzu Nexera X2 UHPLC system (Shimadzu, Kyoto, Japan) equipped with a Raptor ARC-18 column (2.7 μm, 100 × 2.1 mm, Restek, Bad Homburg, Germany) and a Raptor ARC-18 precolumn (2.7 μm, 5 × 2.1 mm, Restek, Bad Homburg, Germany) for separation that was kept at 30°C in the column oven. The mobile phase for the binary gradient containing a mixture of (A) 0.1% formic acid and (B) acetonitrile with 0.1% formic acid at a flow rate of 0.4 mL/min. The gradient was set to 3% B and was raised linearly from 3 B to 10% B within 2.5 min. The gradient of 10% B was held for 2.5 min. Next, the mobile phase was increased to 15% B in 5 min and to 50% in the next 1 min. Subsequently, the mobile phase was held at 50% for 1 min before returning the concentration to 3% for 4 min. The injection volume was 10 μL.

The LC was interfaced with a triple quadrupole ion trap mass spectrometer (LCMS-8050, Shimadzu, Kyoto, Japan) equipped with an ESI source. It operated in the positive mode for all analytes. The ion source parameters were set as follows: heat block, dilution line and interface temperature were set to 400°, 250°, and 300°C, respectively, drying gas, heating gas and nebulizing gas flow were programmed to 10 L/min, 10 L/min, and 3 L/min, respectively, collision-induced dissociation gas was set to 270 kPa, and interface voltage was applied to 4 kV. MS parameters were listed in the previously published paper about the method validation (14). The mass spectrometer was operated in the multiple reaction monitoring (MRM) mode for MS/MS measurements. A waste valve diverted the column effluent to the mass spectrometer just from 2.1 to 7 min to ensure that no salt particles enter the mass spectrometer. Data acquisition was performed with LabSolutions software 5.8 (Shimadzu, Kyoto, Japan). The samples were tested for significance using the *T*-test. The statistical outlier test by Dixon was used for the evaluation of the results.

## Results and discussion

Different durian cultivars from Malaysia, Thailand and Indonesia as well as several other products on the market were analyzed for their folate composition and their total folate content based on fresh weight. More specifically, the aril, which represents the edible part of durian, was analyzed. All samples were determined in duplicates. The results of all analyzed products are given in Table 1. The durian cultivars Golden Phoenix, Black Pearl, Black Thorn, Musang King, Red prawn, King of the King, as well as the popular cultivars D24 and D101 had total folate contents between 307 and 439 μg/100 g. In contrast to this, the moderately smelling cultivar Mon Thong was considerable lower with 175 ± 6.28 μg/100 g. The total folate content of Mon Thong was significantly lower (*p* < 0.05) compared with all other durian varieties analyzed. All samples were analyzed for their vitamers 5-CH_3_-H_4_folate, 5-CHO-H_4_folate, 10-CHO-PteGlu, H_4_folate, and PteGlu generally present in fruits and vegetables. The vitamer distribution of the fresh durian samples was very similar in all samples except of Mon Thong. The main vitamer was shown to be 5-CH_3_-H_4_folate with a percentage of 88 to 92% of the total composition. Minor components were 5-CHO-H_4_folate (1.5–3.9%), and 10-CHO-PteGlu (1.3–3.9%). The most unstable vitamer H_4_folate (1.2–4.7%), and PteGlu (1.6–3.5%) as the fully oxidized vitamer were also present in very low concentrations. Mon Thong with a significantly lower total folate content had a different vitamer composition. 5-CH_3_-H_4_folate is the major vitamer with 47%, however, 5-CHO-H_4_folate and 10-CHO-PteGlu contribute with 19% each to the total folate composition, H_4_folate and PteGlu with 13 and 2.8%.

**Table 1:** Total folate content and vitamer distribution in durian varieties, calculated as PteGlu in [μg/100 g] on fresh weight basis.

	**5-CH_3_-H_4_folate**	**5-CHO-H_4_folate**	**10-CHO-PteGlu**	**H_4_folate**	**PteGlu**	**Total folate**
unknown Durian type	396 ± 10.6	9.96 ± 3.17	17.6 ± 8.07	5.55 ± 0.26	10.7 ± 1.62	440 ± 7.89
Golden Phoenix	286 ± 2.44	12.1 ± 0.16	4.83 ± 0.19	9.68 ± 0.33	9.26 ± 0.26	322 ± 3.38
Black Pearl	276 ± 2.82	6.93 ± 0.05	5.94 ± 0.58	7.37 ± 0.25	10.7 ± 1.41	307 ± 4.62
Black Thorn	316 ± 1.09	6.08 ± 0.13	5.10 ± 0.20	6.36 ± 0.20	9.83 ± 0.20	343 ± 1.54
Musang King	301 ± 8.40	13.0 ± 1.11	5.75 ± 0.32	15.7 ± 2.10	5.22 ± 3.92	340 ± 6.89
Red prawn	363 ± 1.06	11.8 ± 0.21	5.76 ± 0.85	6.96 ± 0.06	11.3 ± 2.29	399 ± 3.92
King of the King	295 ± 1.13	9.37 ± 0.45	5.46 ± 0.51	5.76 ± 0.17	8.71 ± 0.32	324 ± 1.04
D24	286 ± 0.00	7.16 ± 0.10	3.97 ± 0.14	6.79 ± 0.21	7.34 ± 0.20	312 ± 0.03
D101	345 ± 0.21	5.53 ± 0.13	5.72 ± 1.01	7.22 ± 0.28	9.90 ± 0.60	373 ± 0.99
Mon Thong	81.8 ± 5.10	32.9 ± 0.15	33.5 ± 0.00	21.8 ± 0.53	4.87 ± 1.86	175 ± 6.28
Durian seeds	41.9 ± 0.73	7.76 ± 0.95	7.79 ± 2.01	0.49 ± 0.11	n.a.	57.9 ± 3.24
Durian Chips, brand 1	309 ± 9.00	36.8 ± 0.40	14.7 ± 1.40	29.3 ± 0.25	27.1 ± 1.75	417 ± 5.20
Durian Chips, brand 2	35.4 ± 7.91	9.62 ± 0.67	17.3 ± 5.57	1.22 ± 0.08	41.0 ± 16.5	105 ± 20.2
Durian Chips, brand 3	325 ± 22.8	27.6 ± 3.52	4.27 ± 0.96	19.8 ± 3.68	7.44 ± 3.32	384 ± 21.7
Durian Paste	128 ± 18.4	19.6 ± 3.73	3.63 ± 0.79	2.72 ± 1.34	9.25 ± 1.46	163 ± 20.7

Beside the analysis of fresh durian arils, we looked at durian seed (Musang King), which contained 57.9 ± 3.24 μg/100 g on fresh weight basis.

Moreover, we considered regionally popular durian products. We analyzed three commercially available vacuum freeze dried durian Chips, and one durian paste, which were available in most of the durian selling stores. The total folate content is given on the basis of the bought product since this is the way how it is consumed. The durian chips (1–3) revealed to be high in folates with folate contents of 417 ± 5.20 μg/100 g (1), 105 ± 20.2 μg/100 g (2), and 384 ± 21.7 μg/100 g (3). However, considering that the results are based on dried weight basis, the total folate content is significantly lower compared with fresh arils. This might be due to losses during storage. A commercial Mon Thong durian paste showed a total folate of 163 ± 20.7 μg/100 g, however, the paste consists of 90% durian and 10% sugar as well as its preparation involves processing steps such as heating.

The fresh durian arils showed to be tremendously high in folates and therefore a good natural source of folates. The edible aril of popular durian varieties revealed total folates between 307 and 439 μg/100 g. An exception displayed to be the species Mon Thong, which had a much lower content of 175 μg/100 g and a significant different vitamer distribution. These findings correlate well with the recently published draft genome of durian (4). The authors found an upregulated gene expression in sulfur and ripening pathways in Musang King compared with Mon Thong as well as a stronger perceived taste and smell of Musang King cultivars. The latter study reports in arils and particularly in Musang King a higher expression of metabolic genes of methionine, in which folates are essentially involved. Therefore, it can be assumed that the extreme high folate production is necessary for all biosynthesized sulfur-containing odorants. However, this assumption needs to be confirmed in further studies. 5-CH_3_-H_4_folate showed to be the main vitamer in all durian fruits, whereas H_4_folate showed to be a minor component of Durian. The absorption process as well as the post-absorptive metabolism of different folate forms is not well elucidated. Ringling et al. (15) performed a simulation of food folate digestion. Because of its instability, H_4_folate is lost to a high extent during digestion. Therefore, food containing a high proportion of H_4_folate probably has a low bioavailability.

To highlight the folate content of durian, these findings can be compared with other fruits. In a recently published paper (14), we analyzed strawberries and found a total folate content between 59 and 153 μg/100 g. Moreover, Akilanathan et al. (2) investigated diverse fruits commonly consumed in India and found total folates in a large range from 10 to 328 μg/100 g. They found tropical fruits such as Mango between 60 and 138 μg/100 g and bananas between 10 and 188 μg/100 g. Therefore, durian seems to belong to one of the fruits with the highest vitamin B_9_ content.

Reports on folate analysis in Durian are extremely rare, one folate content measured by microbiological assays and listed 2013 in the USDA National Nutrient Database has been removed from the actual version of the latter. Lu et al. (16) analyzed different fruits by microbiological assays and found for durian 225 μg/100 g. However, they did not specify the variety and, therefore, comparison with our results is hardly possible.

## Conclusions

Regarding the estimated consumption of durian arils in Malaysia of 7 g per day per person as outlined in the introduction and assuming a mean folate content in fresh durian arils of 400 μg/100 g, this consumption would equate 28 μg folates. This would cover 9% of the daily intake recommended (300 μg) in Germany, Austria and Switzerland (17). However, as Durian lovers in South East Asia are likely to exceed a daily Durian consumption of 200 g, this could easily cover the daily folate requirements.

Further studies are warranted to provide more detailed information about the folate content and its genetic background in durian. The *Durio* genus includes many different species and we analyzed just few species. Nevertheless, this study contributes to the nutritional clarification and confirms the high economical value of this intriguing exotic fruit.

## Author contributions

LS, YL, DH, and MR conceived and designed the experiments. LS, SC, and CD performed the experiments. LS, YL, DH, and MR analyzed the data and wrote the paper.

### Conflict of interest statement

The authors declare that the research was conducted in the absence of any commercial or financial relationships that could be construed as a potential conflict of interest.
